# SOX2 is essential for *in vivo* reprogramming of seminoma-like TCam-2 cells to an embryonal carcinoma-like fate

**DOI:** 10.18632/oncotarget.9903

**Published:** 2016-06-07

**Authors:** Daniel Nettersheim, Alena Heimsoeth, Sina Jostes, Simon Schneider, Martin Fellermeyer, Andrea Hofmann, Hubert Schorle

**Affiliations:** ^1^ Institute of Pathology, Department of Developmental Pathology, University of Bonn Medical School, Bonn, Germany; ^2^ Institute of Human Genetics, University of Bonn Medical School, Bonn, Germany

**Keywords:** TCam-2, seminoma, embryonal carcinoma, reprogramming, SOX2/SOX17

## Abstract

Type II germ cell cancers (GCC) are divided into seminomas, which are highly similar to primordial germ cells and embryonal carcinomas (EC), often described as malignant counterparts to embryonic stem cells.

Previously, we demonstrated that the development of GCCs is a highly plastic process and strongly influenced by the microenvironment. While orthotopic transplantation into the testis promotes seminomatous growth of the seminoma-like cell line TCam-2, ectopic xenotransplantation into the flank initiates reprogramming into an EC-like fate.

During this reprogramming, BMP signaling is inhibited, leading to induction of NODAL signaling, upregulation of pluripotency factors and downregulation of seminoma markers, like SOX17. The pluripotency factor and EC-marker SOX2 is strongly induced.

Here, we adressed the molecular role of SOX2 in this reprogramming. Using CRISPR/Cas9-mediated genome-editing, we established SOX2-deficient TCam-2 cells. Xenografting of SOX2-deficient cells into the flank of nude mice resulted in maintenance of a seminoma-like fate, indicated by the histology and expression of *OCT3/4, SOX17, TFAP2C, PRDM1* and *PRAME*. In SOX2-deficient cells, BMP signaling is inhibited, but NODAL signaling is not activated. Thus, SOX2 appears to be downstream of BMP signaling but upstream of NODAL activation. So, SOX2 is an essential factor in acquiring an EC-like cell fate from seminomas.

A small population of differentiated cells was identified resembling a mixed non-seminoma. Analyses of these cells revealed downregulation of the pluripotency and seminoma markers OCT3/4, SOX17, PRDM1 and TFAP2C. In contrast, the pioneer factor FOXA2 and its target genes were upregulated, suggesting that FOXA2 might play an important role in induction of non-seminomatous differentiation.

## INTRODUCTION

All invasive type II germ cell cancers (GCC) arise from a common precursor lesion called germ cell neoplasia *in situ* (GCNIS) [1, 2]. However, the GCC subtypes seminoma and embryonal carcinoma (EC) show important differences regarding gene expression, growth and differentiation abilities. While seminomas grow as an undifferentiated cell mass, ECs exhibit features of totipotency and are capable of differentiation into all three germ layers (teratomas) and extra-embryonal tissues (yolk-sac tumor, choriocarcinoma). ECs and seminomas express the pluripotency markers NANOG and OCT3/4, but SOX2 is detected in ECs only and is thought to be compensated by SOX17 in seminomas [3]. In fact, SOX2 and SOX17 serve as markers for diagnostic discrimination between seminomas and ECs.

In a previous study, we demonstrated that xenografting of seminoma-like TCam-2 cells leads to inhibition of BMP signaling, which initiates reprogramming of TCam-2 into an EC-like fate [1, 4, 5]. This reprogramming process was accompanied by strong upregulation of 6 genes classified as initial drivers of the reprogramming process, i. e. *GDF3*, *DPPA3*, *NODAL*, *DNMT3B*, *GAL* and *AK3L1*. The changes in gene expression inversely correlated to the DNA methylation (5mC) levels within corresponding genomic loci [6]. Additionally, many pluripotency and EC associated genes, like *SOX2*, *ZIC3*, *ZFP42*, *LIN28*, *SALL4* and *PRDM14* were upregulated without correlating to changes in their 5mC status [7], while seminoma markers *SOX17*, *cKIT*, *PRDM1* and *PRAME* were downregulated. Once TCam-2 adapted to an EC-like fate, BMP signaling recovered to a level lower than in parental TCam-2 and the newly acquired state was (epi)genetically stabilized by an auto-regulatory NODAL signaling loop and strongly increased 5mC levels, silencing expression of seminoma-associated genes.

As part of the four Yamanaka factors, the pluripotency factor SOX2 is an essential transcription factor for reprogramming of various cells, like fibroblasts to an induced pluripotent state. In murine embryonic stem cells (mESC), Sox2 complexes with Oct3/4 and binds to a canonical motif, thereby driving the expression of pluripotency genes [8]. Overexpression of Sox17 is able to replace Sox2 in the complex with Oct3/4, leading to a change in target site selection to a compressed binding motif [1]. We speculated that during reprogramming of TCam-2 the strong increase in SOX2 protein levels and downregulation of SOX17 leads to a switch to promoters harboring the canonical motif found in pluripotency genes. Furthermore, we postulated that during the seminoma to EC transition, inhibition of BMP signaling leads to derepression of *SOX2*, restoring the classical pluripotency circuitry found in ECs and ESCs, subsequently leading to upregulation of *ZIC3,* which in turn helps to maintain NODAL signaling [7, 9].

In this study, we analyzed the role of the pluripotency factor SOX2 in the *in vivo* reprogramming of TCam-2 to an EC-like cell fate. Therefore, we generated SOX2 knock out TCam-2 cells by utilizing the CRISPR/Cas9 system and xenografted these cells into the flank of nude mice. After six weeks of *in vivo* growth, tumors were isolated and analyzed. Interestingly, TCam-2 cells did not acquire features of an EC, implicating that SOX2 is essential for the transition of TCam-2 cells to an EC-like cell state. Neither upregulation of EC-related pluripotency and epigenetic reprogramming factors, nor induction of NODAL or WNT signaling was detected. Additionally, global 5mC levels remained unaffected and expression of seminoma-associated genes *SOX17*, *PRAME*, *TFAP2C*, *PRDM1* and *cKIT* was maintained. Nevertheless, a small subpopulation initiated differentiation into a mixed non-seminoma, demonstrating that *in vivo* the seminoma-like cell fate cannot be maintained for longer than 6 weeks. This differentiation was accompanied by upregulation of the pioneer factor FOXA2, which interacts with *AFP*, *ALB*, *CDX1*, *DKK1*, *DLK1*, *PITX2*, *TTR*, *EOMES*, apolipoproteins and fibrinogens. So, we hypothesize that this non-seminomatous *in vivo* differentiation of TCam-2 is triggered by FOXA2.

## RESULTS

In a previous study, we demonstrated that TCam-2 cells are able to transit into an EC-like state when being xenografted into the flank of nude mice [10]. Analyses revealed that the somatic microenvironment inhibits BMP signaling, which is the initial step in the reprogramming process of TCam-2 cells, leading to induction of NODAL signaling and expression of EC-related genes as well as globally increased 5mC levels (*in vitro*: 18 %; 6 weeks *in vivo*: 70 %) [10]. During this reprogramming, we found a very rapid (after 1 week of *in vivo* growth) and strong upregulation of the transcription factor SOX2 (Log_2_2.16 fold) and in parallel downregulation of SOX17 (Log_2_3.75 fold) [10]. Due to the importance of SOX2 in cellular reprogramming and maintenance of pluripotency, we were interested in the role of SOX2 in the reprogramming of TCam-2. Therefore, we generated SOX2 knock out TCam-2 clones (TCam-2-ΔSOX2) by utilizing the CRISPR/Cas9 technique. We simultaneously transfected TCam-2 cells with pX330 vector encoding for three different guide RNAs (gRNA) directed towards the SOX2 coding region (Figure S1A). In parallel, a GFP-coding plasmid was transfected to identify clones that presumably have taken up the gRNA-pX330 plasmids. Two days after transfection, single GFP expressing cells were picked and clonally expanded. A PCR analysis revealed that all TCam-2-ΔSOX2 subclones (1 - 5) harbour SOX2 deletions on both alleles and show no band corresponding to the wildtype SOX2 sequence (Figure S1A, S1B). Expression of pluripotency and seminoma key factors was not significantly different between parental TCam-2 and TCam-2-ΔSOX2 clones, suggesting that a CRISPR/Cas9-mediated depletion of SOX2 does not lead to off-target effects, impinging on the undifferentiated and seminoma-like nature of TCam-2 cells (Figure S1C, S1D).

First, we xenografted a TCam-2-ΔSOX2 clone into the flank of nude mice and analyzed the tumor tissue after one week of *in vivo* growth. Macroscopically, the tumor tissues presented as a uniform mass and microscopically showed typical seminoma-morphology i. e. big roundish cells with big nuclei and a clear cytoplasm (Figure S2A, S2B). Additionally, the tumor cells were positive for OCT3/4 and TFAP2C and negative for SOX2 (Figure S2B). By an expression microarray, we analyzed the global gene expression profile of TCam-2-ΔSOX2 cells 1 week after xenografting and TCam-2 *in vitro*. On a global scale, the expression profile of TCam-2-ΔSOX2 cells one week after xenografting and TCam-2 *in vitro* was highly similar (Figure S2C). In more detail, the expression intensities of typical seminoma- and EC-markers (all expressed at very low levels) were highly comparable between xenografted TCam-2-ΔSOX2 cells and TCam-2 *in vitro* suggesting that 1 week after xenografting the TCam-2-ΔSOX2 clones do not differ considerably from *in vitro* cultivated TCam-2 with regard to gene exression (Figure S2D). We found 296 differentially expressed genes (247 upregulated, 49 downregualted). Among the upregulated genes, we found no EC marker and among the downregulated genes, no PGC/GCNIS/seminoma marker was found (Data S1A). Interestingly, among the downregulated genes *ID1* (fold change log_2_1.84) and *ID3* were found, suggesting that similarly to transiting TCam-2, BMP signaling is also inhibited in TCam-2-ΔSOX2 early after xenografting (Data S1A) [10].

**Figure 1:**
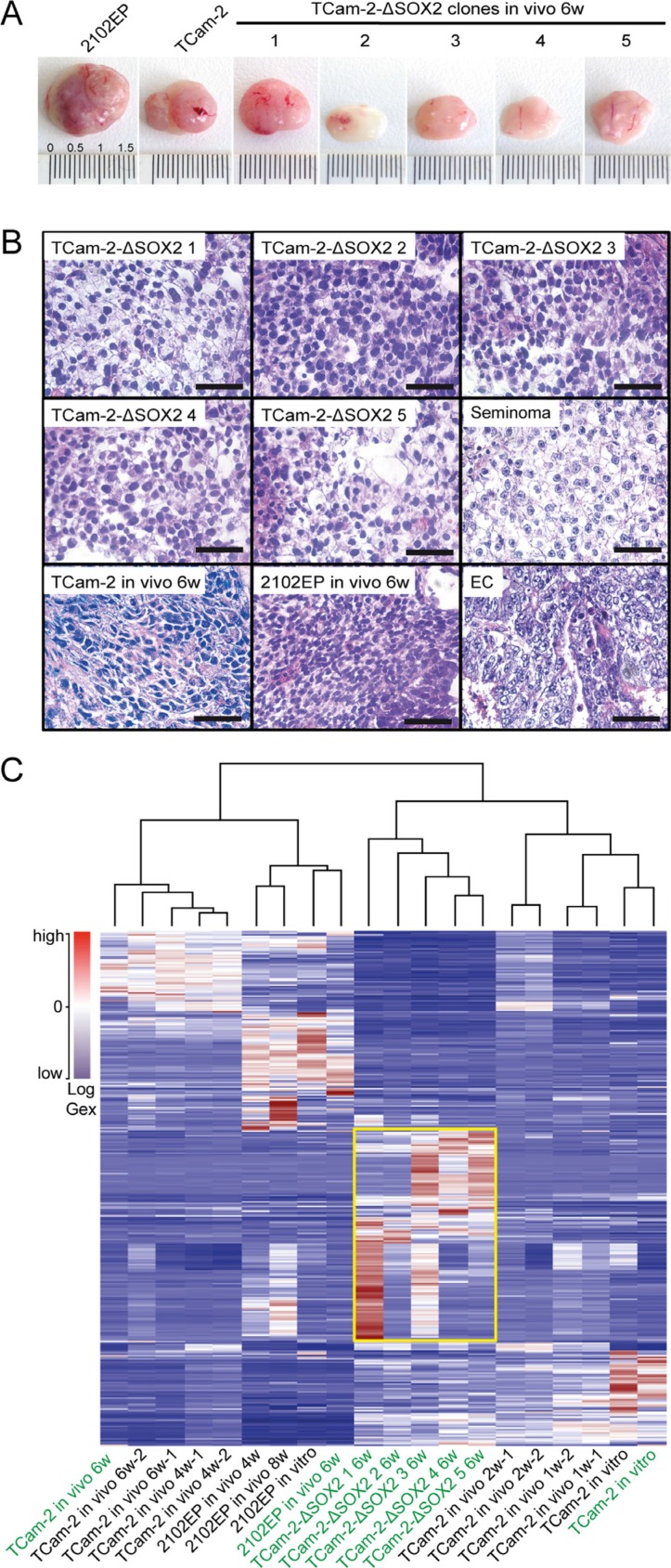
(**A**) Macroscopical appearance of tumors from xenografted TCam-2, 2102EP and TCam-2-ΔSOX2 clones. (**B**) HE staining of tumor tissues from xenografted TCam-2-ΔSOX2 clones, parental TCam-2 and 2102EP. A seminoma and an EC tissue were stained as controls. Scale bars: 50 μm. (**C**) Meta-analysis of expression microarray data of indicated samples including unsupervised hierarchical clustering. Samples labelled in green were generated for this study, samples in black are part of a previous publication [10] and re-analyzed in context of this study. The yellow box highlights genes differentially expressed between TCam-2-ΔSOX2 clones and the other samples.

Next, we asked if the TCam-2-ΔSOX2 clones maintain their seminomatous nature for longer than 1 week *in vivo*. Therefore, we xenografted the 5 TCam-2-ΔSOX2 clones into the flank of nude mice and monitored tumor growth for six weeks. As controls, parental TCam-2 and 2102EP were transplanted. 2102EP cells express typical EC- and pluripotency factors, but are nullipotent and thus less prone to differentiation than other EC cell lines. So, 2102EP cells closely resemble an undifferentiated EC *in vitro* and *in vivo*. After six weeks, tumors were recovered and analyzed. Macroscopically, the 2102EP grew into the largest tumor, followed by the TCam-2 tumor, which should have adapted an EC-like cell fate at this time point (Figure 1A) [10]. One TCam-2-ΔSOX2 tumor (1) showed a similar size as the TCam-2 tumor, while TCam-2-ΔSOX2 tumors 2 - 5 were considerably smaller (Figure 1A). In contrast to the 2102EP and TCam-2 tumor, the TCam-2-ΔSOX2 tumors appeared as a homogeneous mass (Figure 1A). HE staining of TCam-2-ΔSOX2 tumors revealed a typical seminoma-like morphology (Figure 1B). In contrast, tumors from xenografted 2102EP and TCam-2 cells displayed a typical EC morphology, i. e. small polygonal and eosinophilic cells with hard to distinguishable cellular borders (Figure 1B).

To analyze genome-wide deregulation in gene expression, we performed microarray analyses of the TCam-2-ΔSOX2 tumors. Tumors from xenografted TCam-2 and 2102EP cells as well as *in vitro* cultivated TCam-2 served as control. We compared the global expression profiles using unsupervised hierarchical clustering and illustrated the data as a heatmap (samples labeled in green) (Figure 1C). Additionally, we meta-analyzed microarray expression data from a previous study elucidating the reprogramming of TCam-2 to an EC-like fate *in vivo* (all samples labeled in black) [10]. The hierarchical clustering and heatmap analysis demonstrated that all five TCam-2-ΔSOX2 clones clustered closely to each other, demonstrating good reproducibility of the analysis. Further, the tumor from xenografted 2102EP clustered to the 2120EP *in vitro* and *in vivo* samples from our previous study and the tumor samples from xenografted TCam-2 clustered to the tumor tissues from TCam-2 after 4 and 6 weeks of *in vivo* growth. Finally, our TCam-2 *in vitro* control clustered to our TCam-2 control from our previous study and to TCam-2 cells grown *in vivo* for 1 and 2 weeks, which are still closely related to TCam-2 *in vitro*. Interestingly, the TCam-2-ΔSOX2 clones clustered more closely to the TCam-2 control cells (1 w, 2 w, *in vitro*) than to the 2102EP (4 w, 8 w, *in vitro*) or reprogrammed TCam-2 samples (4 w, 6 w). Thus, the TCam-2-ΔSOX2 clones display a global gene expression profile more similar to a seminoma than an EC. Nevertheless, the TCam-2-ΔSOX2 clones deregulated a cluster of genes, which is not altered in all other analyzed samples (Figure 1C, yellow box), indicating that the TCam-2-ΔSOX2 clones also underwent changes in gene expression.

**Figure 2:**
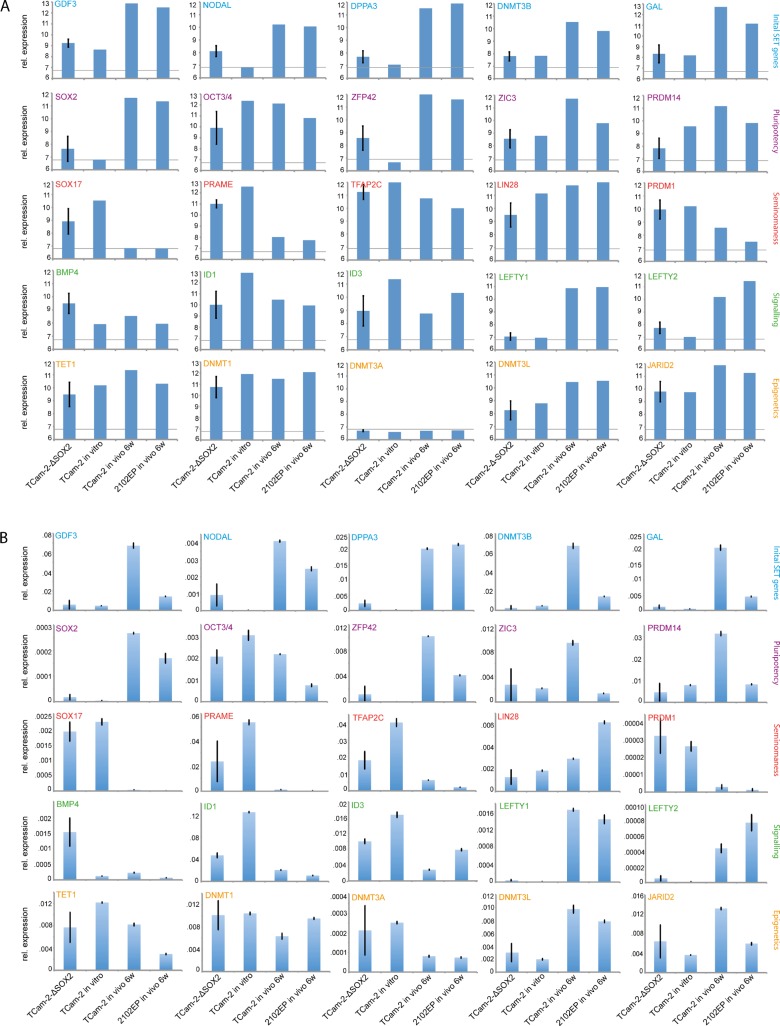
(**A**, **B**) Expression microarray (A) and qRT-PCR (B) data of indicated genes related to the *in vivo* reprogramming, pluripotency, seminoma-ness, signaling pathways and epigentics. For TCam-2-ΔSOX2 clones, expression data of five clones was averaged. Standard deviation is indicated above each bar. Genes showing expression values below the grey line in (A) were considered as not expressed (Log_2_6.8).

Next, we screened the microarray data for expression of key factors driving the seminoma to EC transition, pluripotency factors, seminoma markers, signaling pathway-related genes and epigenetic factors (Figure 2A, Data S1B) [10]. qRT-PCR analyses verified all detected deregulation in gene expression (Figure 2B). In contrast to xenografted TCam-2 and 2102EP cells, the TCam-2-ΔSOX2 clones showed no upregulation of the initial reprogramming genes *GDF3*, *NODAL*, *DPPA3*, *DNMT3B* and *GAL* [10] (Figure 2A, 2B). *SOX2* was not significantly upregulated in TCam-2-ΔSOX2 clones compared to TCam-2 *in vitro*. Other pluripotency factors were either slightly down- (*OCT3*/*4, PRDM14)* or upregulated (*ZFP42*) or remained unchanged (*ZIC3*) compared to *in vitro* cultivated TCam-2, but expression intenstities were clearly different to xenografted TCam-2 or 2102EP. In TCam-2-ΔSOX2 clones, expression of seminoma markers *SOX17*, *PRAME*, *TFAP2C*, *LIN28* and *PRDM1* was slightly downregulated or remained unchanged compared to the TCam-2 *in vitro* cells. Initially during the seminoma to EC transition, BMP signaling is inhibited, indicated by strong downregulation of *ID1*/*3* [10]. At late stages of the reprogramming, BMP signaling is re-activated by upregulating *BMP4* [10]. Additionally, expression of *ID1*/*3* recovers to a level lower than in parental TCam-2 cells [10]. Inhibition of BMP signaling leads to activation of NODAL signaling [10]. In TCam-2-ΔSOX2 clones, 1 and 6 week(s) after xenografting we found downregulation of *ID1*/*3* to a level comparable to xenografted TCam-2/2102EP (Data S1C; Figure 2A, 2B). This suggests that during *in vivo* growth of TCam-2-ΔSOX2 clones, like during reprogramming of parental TCam-2, BMP signaling is inhibited and remains constantly low compared to parental TCam-2 cells [10]. Nevertheless, *NODAL*, *LEFTY1* and *LEFTY2* were not significantly upregulated, indicating inactive NODAL signaling and suggesting that NODAL signaling is regulated by SOX2.

**Figure 3:**
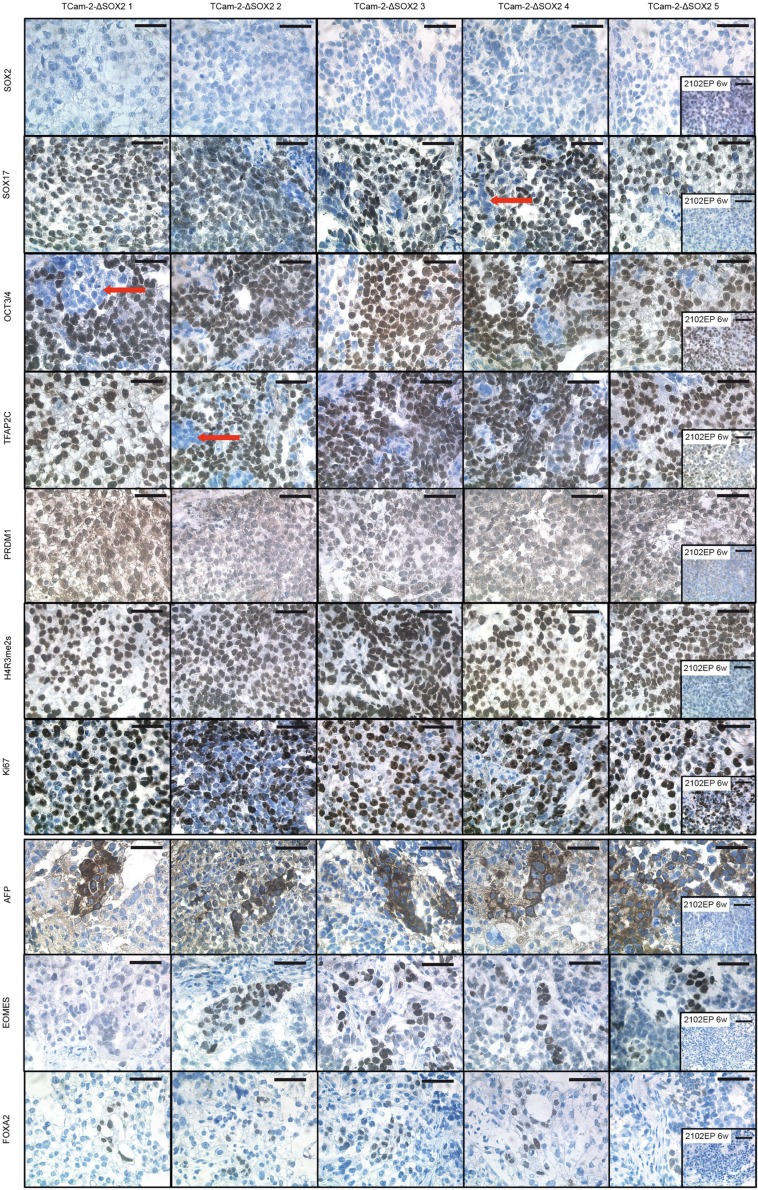
IHC staining of indicated proteins in TCam-2-ΔSOX2 clones Tumor tissues from xenografted 2102EP cells were stained as control. Scale bars: 50 μm.

IHC staining confirmed absence of SOX2 and expression of SOX17, OCT3/4 and TFAP2C in all xenografted TCam-2-ΔSOX2 clones (Figure 3). Furthermore, IHC demonstrated nuclear localization of PRDM1, which is a hallmark of GCNIS/seminomas, while in ECs PRDM1 is located in the cytoplasm [11] (Figure 3). In murine PGCs, nuclear Prdm1 establishes in concert with Prmt5 a symmetric dimethylation of arginine 3 on histones H2A and H4 (H2A/H4R3me2s), which suppresses somatic differentiation programs [12, 13]. Accordingly, H4R3me2s was strongly detectable in TCam-2-ΔSOX2 clones, but not in 2102EP cells (Figure 3). This suggests that in PRDM1/H4R3me2s positive TCam-2-ΔSOX2 cells, somatic differentiation is epigenetically blocked, contributing to maintenance of a seminoma-like cell fate. A strong Ki67 signal indicated a high proliferative activity of the TCam-2-ΔSOX2 tumor cells (Figure 3).

**Figure 4:**
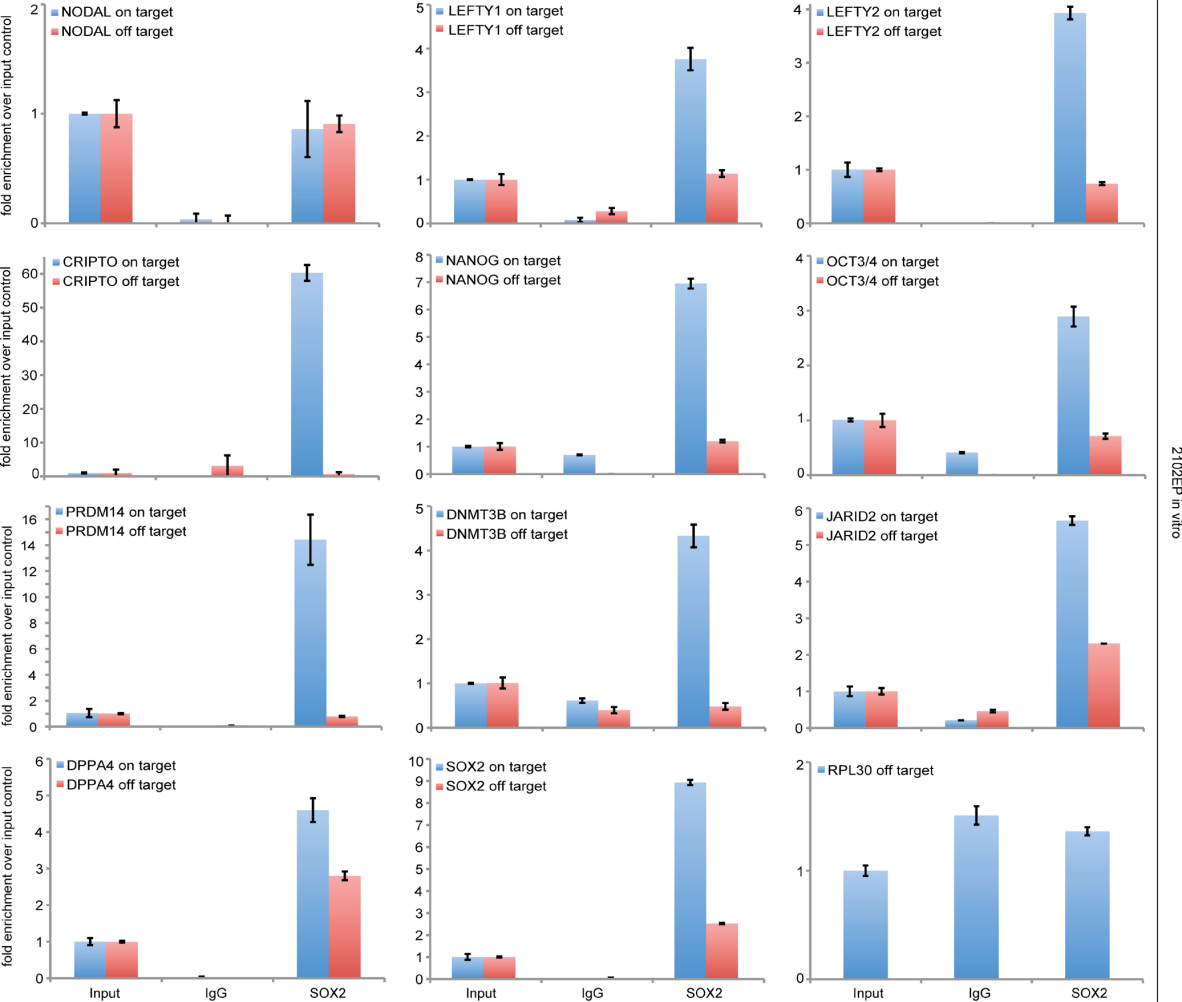
ChIP followed by qPCR-analysis of SOX2-enrichment at the SOX2 binding sites within indicated genes 3% input chromatin was used for normalization and an IgG antibody was used as negative ChIP control. In qPCR, oligonucleotides were used to amplify a PCR-fragment around the SOX2 binding site (on target) and a PCR-fragment within the same gene, but without a SOX2 binding site (off target).

Our data suggested that NODAL signaling might be regulated by SOX2. Thus, we analyzed 2102EP cells as a proxy for an EC by chromatin-immunoprecipitation (ChIP) using a SOX2 antibody followed by qPCR-analysis. We detected enrichment of SOX2 at the promotors of the NODAL signaling key factors *LEFTY1/LEFTY2* and *CRIPTO*, which contain SOX2 binding sites (Figure 4) [14]. *NODAL* itself was not enriched over input control (Figure 4). As positive controls, we analyzed the promoters of *SOX2*, *NANOG* and *OCT3*/*4* and confirmed enrichment of SOX2 at these elements (Figure 4) [15–18]. All genes analyzed are also expressed in 2102EP/EC cells [10, 19]. As a negative control, the promoter of the *RPL30* Gene was analyzed, where we did not detect enrichment of SOX2 (Figure 4). Thus, it is highly likely that during *in vivo* growth of TCam-2-ΔSOX2 clones NODAL signaling remains inactive, because induction of the essential NODAL co-factor *CRIPTO* and *LEFTY1*/*2* and subsequently establishment of the NODAL signaling loop requires SOX2.

We asked, if other factors that contain a SOX2 binding site and are involved in reprogramming of TCam-2 to an EC might be bound and regulated by SOX2. Again, we analyzed 2102EP cells as a proxy for an EC by SOX2-ChIP and found enrichment of SOX2 at corresponding binding sites within the promoters of *DNMT3B*, *PRDM14*, *JARID2* and *DPPA4* (Figure 4). The fact that these factors are strongly upregulated during the seminoma to EC transition of TCam-2, suggests that SOX2 induces expression of these genes [10].

**Figure 5:**
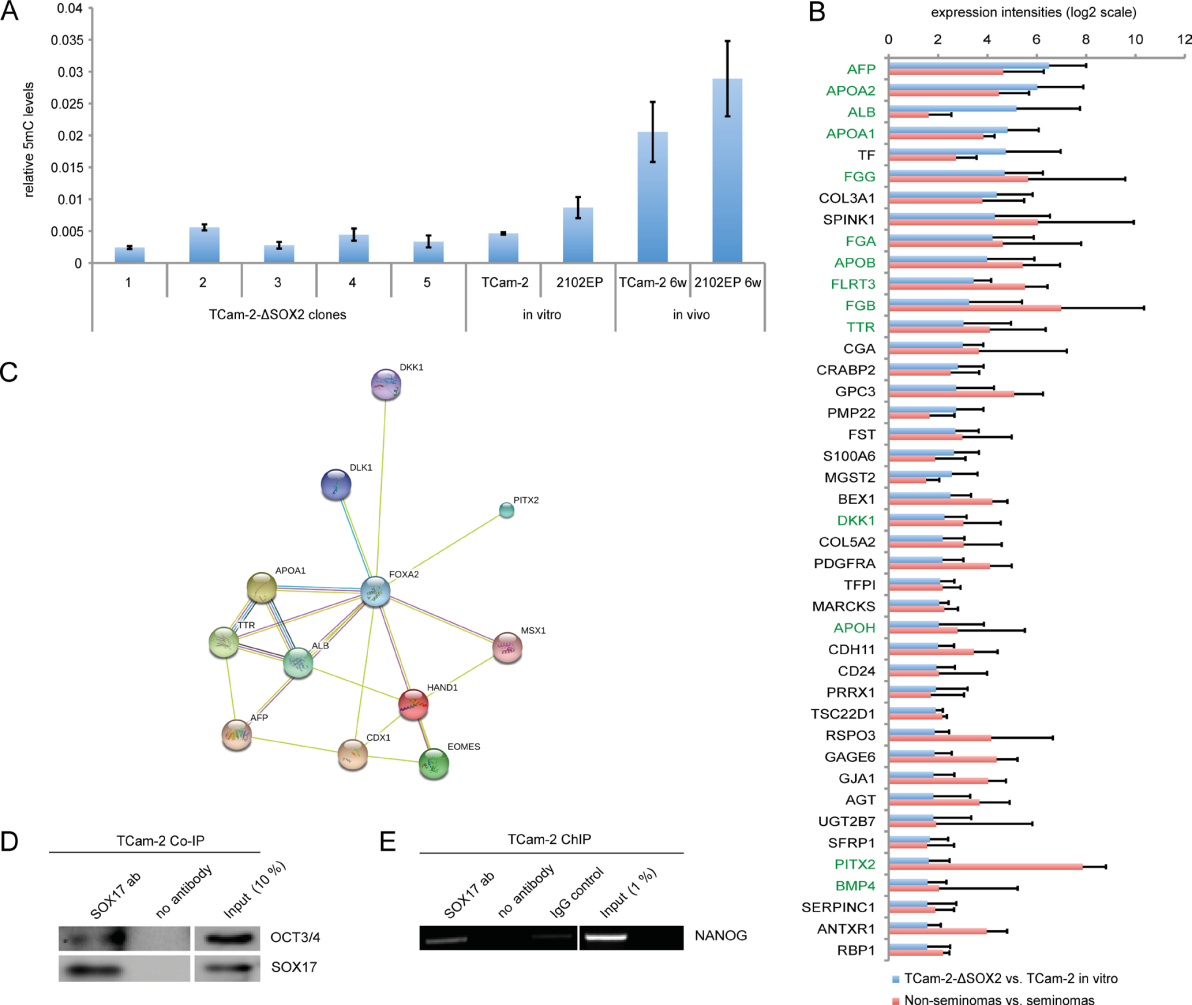
(**A**) Quantification of 5mC levels in tumors from xenografted TCam-2-ΔSOX2 clones, TCam-2 and 2102EP cells. In vitro cultivated TCam-2 and 2102EP served as controls. (**B**) Commonly expressed genes in TCam-2-ΔSOX2 (vs. TCam-2 *in vitro*) and non-seminomas (*n* = 7) (vs. seminomas (*n* = 4)) based on microarray data. Green labeled genes are related to FOXA2. (**C**) STRING analysis of FOXA2 interacting genes. (**D**) Co-IP experiment in TCam-2 demonstrating binding of SOX17 to OCT3/4. (**E**) ChIP experiment in TCam-2, demonstrating binding of SOX17 to the SOX2 / OCT3/4 binding motif within the NANOG promoter.

The seminoma to EC transition is accompanied by upregulation of the de novo DNA methyltransferase *DNMT3B*, resulting in a strong increase in DNA methylation levels [8, 10]. In TCam-2-ΔSOX2 cells, *DNMT3B* is not upregulated and the expression levels of other epigenetic regulators (*TET1*, *DNMT1*, *DNMT3A*, *DNMT3L*, *JARID2*) are highly comparable to *in vitro* cultivated TCam-2 (Figure 2A, 2B). Thus, depletion of *SOX2* prevents the induction of *DNMT3B* and other epigenetic factors. In line, global DNA methylation levels do not increase in TCam-2-ΔSOX2 clones, but in xenografted TCam-2 cells that adapted to an EC-like fate and 2102EP cells (Figure 5A).

In summary, in SOX2 depleted clones expression of seminoma markers is maintained and the initial reprogramming markers, pluripotency genes and epigenetic factors are not induced. Additionally, although BMP signaling seems to decrease and recover during *in vivo* growth of the TCam-2-ΔSOX2 clones, NODAL signaling is not activated, suggesting that SOX2 controls activation of NODAL signaling. Further, expression of epigenetic factors, including de novo DNA methyltransferases and DNA methylation levels remain at the level of TCam-2. Thus, the data demonstrate that inhibition of BMP signaling does not depend on SOX2. Interestingly, the lack of NODAL activation strongly suggests that SOX2 is essential for this step. Hence, SOX2 acts downstream of BMP signaling and upstream of the NODAL cascade.

Although the vast majority of cells stained positive for the seminoma markers SOX17, OCT3/4 and TFAP2C, small areas displayed absence of staining of these pluripotency- and seminoma-related genes, pointing at a heterogeneity of the tumor tissues (Figure 3, red arrows). We hypothesize that this subpopulation represents a differentiated population.

In a previous study, we demonstrated that TCam-2 cells directly differentiate *in vitro* into a cell type resembling a mixed non-seminoma, when being cultivated in fibroblast-conditioned medium supplemented with FGF4/heparin [20]. During this differentiation, an EC-intermediate step is skipped (no upregulation of SOX2) and a strong upregulation of germ layer differentiation markers, like *AFP* (also a yolk-sac tumor marker) and *HAND1* was observed [20]. *SOX17* expression was downregulated [20]. Morphologically, the cells presented as large roundish cells with a big nucleus [20]. Additionally, the cells showed features of trophectodermal/choriocarcinoma-like cells, i. e. presence of multinucleiated cells and upregulation of *GATA2*, *GATA6*, *TEAD1* and *TEAD4* [20]. Pluripotency and seminoma markers like *NANOG*, *OCT3*/*4*, *LIN28*, *TFAP2C*, *cKIT*, *D2-40* and *PRDM1* were downregulated [20]. Downregulation of PRDM1 was accompanied by reduced levels of H2a/H4R3me2s, allowing for differentiation [20].

We repeated this *in vitro* differentiation experiment and included the TCam-2-ΔSOX2 clones (Figure S2E). Under *in vitro* differentiating conditons both, the parental and SOX2-deficient TCam-2 cells downregulated pluripotency/seminoma markers *OCT3*/*4*, *TFAP2C*, *PRDM14* and *PRAME,* while *SOX2* and *DNMT3B* expression remained low (Figure S2F). In contrast, expression of the trophoblast stem cell marker *EOMES* and germ layer differentiation markers *AFP*, *HAND1, FOXA2* (endoderm) and *PAX6*(ectoderm) were strongly upregulated (Figure S2F). *SOX17* remained expressed, but to a level considerably lower than under non-differentiating conditions (Figure S2F) [20]. Similar to the *in vivo* growth of TCam-2-ΔSOX2 cells, *NODAL*, but not *LEFTY1*/*LEFTY2* or *CRIPTO* was upregulated under differentiating conditions, further strengthening the notion that SOX2 is necessary to build up a functional NODAL signaling loop (Figure S2F). In summary, the deficiency of SOX2 does not impair the *in vitro* differentiation process of TCam-2 cells into a cell type resembling a mixed non-seminoma, further demonstrating that no EC intermediate state is necessary.

We screened the microarray data of TCam-2-ΔSOX2 for deregulations in gene expression indicative for a differentiation process similar to the *in vitro* differentiation of TCam-2. From all genes differentially expressed between TCam2-ΔSOX2 clones (6 w) and TCam-2 *in vitro*, we found 124 probes coding for 112 annotated genes significantly (fold change ≥ Log_2_1.5) upregulated and 500 probes (437 annotated genes) downregulated (Data S1 B). In the TCam-2-ΔSOX2 clones, we found very similar results as during the *in vitro* differentiation of TCam-2, i. e. upregulation of endodermal (*AFP*, *FOXA2*, *CDX1*), mesodermal (*HAND1*, *PRRX1*, *PDGFRA*, *GJA1*, *PITX2*, *CXXC5*, *DKK1*, *AIF1*, *MSX1*, *GPC3*, *DACT3, HOXC8*) and ectodermal (*SOX11*, *DLK1*, *BEX1*) differentiation markers as well as downregulation of pluripotency factors *OCT3/4* and *LIN28* (Data S1B). Additionally, the trophoblast stem cell marker *EOMES* and syncytiotrophoblast-associated chorionic gonadotropins *CGB1*, *CGB5, CGB8* were upregulated in TCam-2-ΔSOX2 clones compared to TCam-2 *in vitro* (Data S1B). By IHC, we confirmed upregulation of AFP, FOXA2 and EOMES in a subset of cells morphologically appearing differentiated within TCam-2-ΔSOX2 tumor tissues (Figure 3). Additionally, qRT-PCR analysis validated upregulation of *AFP* and *HAND1* in TCam-2-ΔSOX2 (Figure S3A).

Within the OCT3/4/SOX17/TFAP2C negative subpopulation, very large roundish cells with a big nucleus (green arrows) and multinucleated cells (red arrows) were found (Figure S3 B). Furthermore, PRDM1 was excluded from the nucleus in these cells (Figure S3 B, blue arrows). We postulate that the small subpopulation of OCT3/4/SOX17/TFAP2C negative cells is highly similar to *in vitro* differentiated TCam-2 and thus resembles a mixed non-seminoma *in vivo*.

We asked, if first signs of a differentiation process into a mixed non-seminoma can be found already one week after xenografting. Therefore, we compared all genes deregulated in TCam-2-ΔSOX2 clones 6 weeks after xenografting to genes deregulated one week after xenografting (fold change ≥ Log_2_2). We found only 14 genes commonly upregulated and 11 downregulated (Data S1 C). The upregulated genes were not indicative for a differentiation process and from the downregulated genes no loss of seminona-like fate could be concluded. Although, *ID3* was among the set of downregulated genes, pointing at inhibited BMP signaling as already discussed. In conclusion, differentiation into a mixed non-seminoma is initiated later than one week after xenografting.

To support our hypothesis of a mixed non-seminoma-like differentiation process, we compared the deregulations found in the TCam-2-ΔSOX2 clones to differences in gene expression between non-seminoma and seminoma tissues (fold change ≥ Log_2_1.5) gained from microarray data published previously [10, 21] (Data S1 D). From the 122 genes found to be upregulated in TCam-2-ΔSOX2 clones versus TCam-2 *in vitro*, 42 genes were also upregulated in non-seminomas versus seminomas (Figure 5B, Data S1D). Among them, differentiation markers like *AFP*, *ALB*, *DKK1*, *FST*, *GJA1*, *PITX2*, *GPC3*, *CGA* and several apolipoproteins and fibrinogens (Figure 5B). This suggests that the cluster of genes upregulated during *in vivo* differentiation of TCam-2-ΔSOX2 cells reflects differences in expression between non-seminomas and seminomas, confirming our postulated non-seminomatous differentiation process.

In TCam-2-ΔSOX2 cells, we found a strong upregulation of *FOXA2*, a pioneer factor able to open compacted chromatin and regulator of expression in differentiated tissues and during embryonic development [22–24]. FOXA2 has been described as a transcriptional activator for AFP and albumin (ALB) and regulator of lipid metabolism and fibrinogens [25–27]. Furthermore, FOXA2 is involved in development of endodermal-derived organs like the liver, pancreas and lungs [28, 29]. In TCam-2-ΔSOX2 clones, we found a strong upregulation of FOXA2 target genes *AFP* and *ALB* (Data S1B). Additionally, lipid metabolism associated apolipoproteins (*APOA1*, *APOA2*, *APOB*, *APOC1*, *APOE*, *APOH*, *APOM*), fibrinogens and related factors (*FGA*, *FGB*, *FGG*, *FGL1*, *FLRT3*) as well as several endodermal factors (*HPX, CDX1)* were upregulated (Data S1B). STRING analysis of all genes upregulated in TCam-2-ΔSOX2 clones versus TCam-2 *in vitro* predicted interaction of FOXA2 with many of these genes (Data S1E). Among the genes commonly expressed in TCam-2-ΔSOX2 clones (vs. TCam-2 *in vitro*) and in non-seminomas (vs. seminomas) are several FOXA2 targets (Figure 5B, green labeled genes; Data S1E). Thus, in TCam-2-ΔSOX2 clones *FOXA2* might be an important driver of somatic differentiation.

## DISCUSSION

In this study, we generated SOX2-deficient TCam-2 cells by the CRISPR/Cas9 technique and xenografted these cells into the flank of nude mice. Molecular analyses of the tumors demonstrated a seminoma-like morphology and gene expression profile, suggesting that SOX2 is essential for induction of an EC-like cell fate. In TCam-2-ΔSOX2 clones, BMP signaling related molecules *ID1* and *ID3* were downregulated, which is indicative for reduced BMP signaling activity. However, activation of NODAL (early during reprogramming of TCam-2) and WNT (late during reprogramming) signaling and upregulation of pluripotency and EC markers *GDF3*, *DPPA3*, *DNMT3B*, *ZIC3*, *PRDM14* failed to occur [10]. Thus, SOX2 appears to be downstream of BMP signaling but upstream of NODAL activation. Our ChIP analysis in 2102EP suggested that SOX2 regulates expression of *LEFTY1/LEFTY2* and *CRIPTO*, allowing for establishment of the NODAL signaling loop. *In vitro* treatment of TCam-2 cells (SOX2 negative) with recombinant NODAL was not sufficient to activate NODAL signaling [10]. So, expression of SOX2 is a prerequisite for activation of NODAL signaling. Furthermore, our ChIP experiments demonstrated binding of SOX2 to key factors of pluripotency and ECs (*SOX2*, *DPPA3*, *DNMT3B*, *PRDM14*, *GDF3*). In conclusion, SOX2 is an essential factor in acquiring the EC-like cell fate from GCNIS or seminoma.

Most importantly, the majority of TCam-2-ΔSOX2 cells maintains a seminoma-like cell fate for at least 6 weeks *in vivo*, indicated by a typical seminoma-like morphology and expression of seminoma markers *SOX17*, *PRAME*, *TFAP2C* and *PRDM1* (nuclear). Nevertheless, we observed an OCT3/4, SOX17 and TFAP2C negative subpopulation showing cytoplasmic PRDM1 and lack of H4R3me2s. Additionally, microarray analyses revealed upregulation of markers indicative for differentiation into all three germ layers, like *AFP*, *HAND1* and *CDX1*. This expression profile is highly similar to *in vitro* differentiated TCam-2 cells that resemble a mixed non-seminoma [20]. There, the differentiated cells also upregulated *AFP* and *HAND1* and showed nuclear exclusion of PRDM1. AFP is a strong marker for the non-seminomatous GCC entity yolk-sac tumor. Additionally, cells appeared morphologically as big roundish cells with a big nucleus - a morphology also found in the OCT3/4/SOX17/TFAP2C negative subpopulation of TCam-2-ΔSOX2 tumors. Furthermore, *in vitro* differentiated TCam-2 gained features of trophoblast cells, like upregulation of *TEAD4* and formation of syncytiotrophoblastic multinucleated cells. We also found multinucleated cells within the differentiated subpopulation of *in vivo* grown TCam-2-ΔSOX2 cells as well as upregulation of the trophoblast stem cell marker *EOMES*. So, the OCT3/4/SOX17/TFAP2C negative subpopulation cleary recapitulates the *in vitro* differentiation process of TCam-2 cells into a mixed non-seminoma *in vivo*. As *in vitro*, an EC intermediate is skipped. Thus, we showed for the first time that TCam-2 cells are able to directly differentiate into a mixed non-seminoma *in vivo*. This development of a mixed non-seminomatous tumor does not require a SOX2-positive EC intermediate.

How is the differentiation process initiated? In murine ESCs, SOX2 promotes pluripotency, while SOX17 drives differentiation into endodermal lineage. Both, SOX2 and SOX17 are able to partner with OCT3/4, but different binding motifs are occupied, regulating expression of pluripotency- and self-renewal-associated factors (SOX2/OCT3/4) or differentiation-related genes (SOX17/OCT3/4). In human seminomas/TCam-2 cells, SOX17 and OCT3/4 are expressed, but endodermal differentiation programs are suppressed. We demonstrated that in TCam-2, SOX17 co-immunoprecipitates with OCT3/4 and is able to bind to the NANOG promotor containing a SOX2/OCT3/4 binding motif (Figure 5D, 5E). Thus, in undifferentiated seminomas it is highly likely that SOX17 is redundant to SOX2 and promotes expression of pluripotency factors in combinaton with OCT3/4, thereby maintaining a GCNIS/seminoma-like cell fate.

During reprogramming of TCam-2, SOX17 is replaced by SOX2, leading to acquisition of an EC-like fate. In TCam-2-ΔSOX2 cells, the switch from SOX17 to SOX2 is not possible and prolonged expression of SOX17/OCT3/4 contributes to maintenance of a seminoma fate.

In the differentiated subpopulation of *in vivo* grown TCam-2-ΔSOX2 cells, we found a strong upregulation of FOXA2, a pioneer factor, which is able to open compacted chromatin and regulate gene expression in differentiated tissues and during embryonic development [22, 23, 24]. Additionally, several FOXA2 target genes were upregulated in TCam-2-ΔSOX2 clones. Thus, *FOXA2* might be an important factor in promoting differentiation of the TCam-2-ΔSOX2 cells into a mixed non-seminoma. In line, the FOXA2 protein was detectable only in OCT3/4/SOX17/TFAP2C negative cells, which appear morphologically as differentiated.

Interestingly, the FOXA2-driven differentiation seems to be independent of SOX17, since the SOX17 protein is not detectable in differentiated TCam-2-ΔSOX2 subpopulation. Thus, in contrast to the murine system, SOX17 presumably is not involved in the differentiation of human seminomas into a cell type resembling a mixed non-seminoma.

Together with the protein arginine methyltransferase PRMT5, nuclear PRDM1 dimethylates arginine 3 on the histones H2A and H4. By this epigenetic mechanism, somatic differentiation programs are suppressed in murine PGCs [12, 13]. Nuclear PRDM1 and the H2A/H4R3me2s was also found in human seminomas, but not in ECs [11]. During *in vitro* differention of TCam-2 into a mixed non-seminoma and during the *in vivo* reprogramming, PRDM1 is downregulated and excluded from the nucleus [10, 20, 30]. Thus, in TCam-2-ΔSOX2 clones, nuclear PRDM1 might be responsible for maintenance of H2A/H4R3me2s and suppression of somatic differentiation. In contrast, in the OCT3/4/SOX17/TFAP2C negative subpopulation nuclear exclusion of PRDM1 leads to reduced H2A/H4R3me2s levels, putatively enabling differentiation.

**Figure 6:**
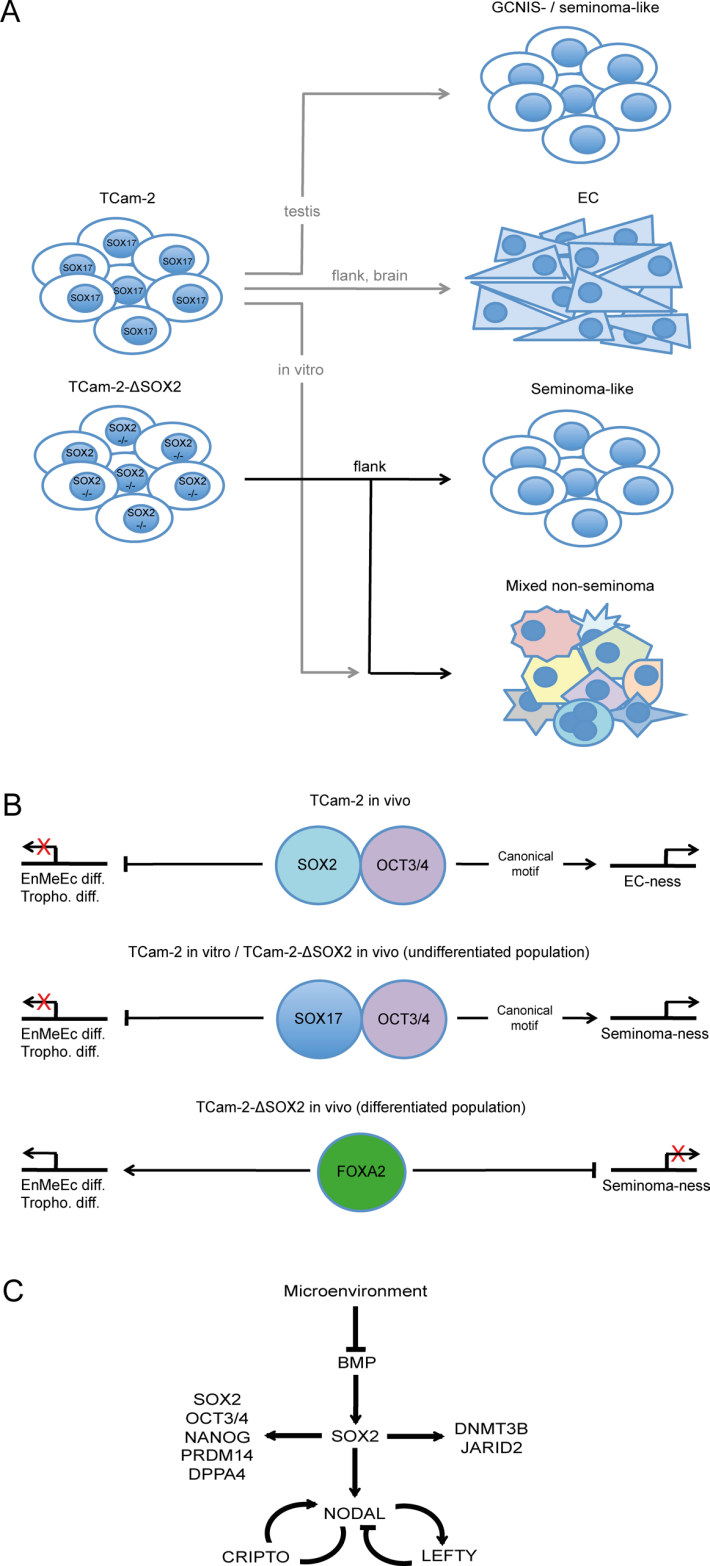
(**A**) Model of the differentiation abilities of TCam-2 and TCam-2-ΔSOX2 cells *in vitro* and *in vivo*. (**B**) Molecular mechanisms of promoting EC- or seminoma-ness and differentiation into endodermal-, mesodermal- and ectodermal-lineage (EnMeEc diff.) and trophectoderm (Troph. diff.). (**C**) Detailed mechanism of the role of SOX2 in reprogramming of TCam-2 to an EC-like state *in vivo*. Models are based on the results of this study and [8, 10, 20, 30].

In summary, TCam-2 grow as seminoma-like in the testis and transit into an EC-like fate when being xenografted into the somatic microenvironment of the murine flank or brain (Figure 6A) [8, 10, 30]. Additionally, TCam-2 can be forced to differentiate into a mixed non-seminoma upon cultivation of murine fibroblast conditioned medium supplemented with FGF4 (Figure 6A) [20]. TCam-2-ΔSOX2 maintain a seminoma-like fate *in vivo* for at least 6 weeks, but initiation of differentiation in a subpopulation cannot be prevented (Figure 6A). This differentiation is highly similar to the *in vitro* differentiation into a mixed non-seminoma. Recently, a study found high intratumoral heterogeneity in GCC tissues from about 615 patients [31]. There, patients suffering from a mixed yolk-sac-seminoma tumor had the poorest clinical outcome [31]. In our study, strong upregulation of AFP in the OCT3/4 negative TCam-2-ΔSOX2 subpopulation is indicative for development of a yolk-sac tumor. Thus, 6 weeks after xenografting a yolk-sac-seminoma tumor component is present. So, upon contact with a somatic microenvironment, seminomas may develop into more aggressive yolk-sac-seminoma-like tumors that need different therapeutic strategies as classical seminomas. Tu et al. propose that an integrated or multimodal therapy may be effective at addressing intratumoral heterogeneity and treating distinct subtypes as well as a potentially lethal phenotype of non-seminomatous GCCs [31]. Xenografting of TCam-2-ΔSOX2 cells provides a useful model to adress these issues regarding mixed yolk-sac-seminoma-like tumors.

We have shown evidence that upon xenografting of TCam-2 cells into the flank SOX17 is replaced by SOX2 [8] [10]. Based on our previous published data and this study, we propose that the somatic microenvironment inhibits BMP signaling, leading to upregulation of SOX2, which partners with OCT3/4. Together, SOX2 and OCT3/4 drive acquisition of an EC-like fate by contributing to establishment of the NODAL signaling loop (*LEFTY1*/*2*, *CRIPTO*) and regulating pluripotency- (*SOX2*, *OCT3*/*4*, *NANOG*, *PRDM14*, *DPPA4*) and epigenetic reprogramming-factors (*DNMT3B*, *JARID2*) (Figure 6B, upper panel; C). In seminoma-like TCam-2-ΔSOX2 cells SOX17 expression is maintained and presumably partners with OCT3/4 to bind the canonical motif, triggering expression of PGC-, GCNIS- and seminoma-related genes, allowing keeping up the seminoma-like cell fate (Figure 6B, middle panel). Nevertheless, differentiation into a mixed non-seminoma is not blocked in TCam-2-ΔSOX2 cells. We hypothesize that in this OCT3/4/SOX17 negative subpopulation the pioneer factor FOXA2 initiates differentiation into endodermal-, mesodermal- and ectodermal-lineage as well as trophectoderm (Figure 6B, lower panel).

## MATERIALS AND METHODS

### Ethics statement

All animal experiments were conducted according to the German law of animal protection and in agreement with the approval of the local institutional animal care committees (Landesamt für Natur, Umwelt und Verbraucherschutz, North Rhine-Westphalia (approval ID: AZ-84-02.04.2013-A430). The experiments were conducted in accordance with the International Guiding Principles for Biomedical Research Involving Animals as announced by the Society for the Study of Reproduction.

### Cell culture

GCC cell lines utilized in this study were cultivated as described previously [10]. Briefly, TCam-2 cells (provided by Dr. Janet Shipley, Institute of Cancer Research, Sutton, UK) were grown in RPMI, 2102EP and NCCIT (both from Prof. Dr. Leendert Looijenga, Erasmus MC, Daniel den Hoed Cancer Center, Josephine Nefkens Institute, Rotterdam, NL) in DMEM. *In vitro* differentiation of TCam-2 and TCam-2-ΔSOX2 was induced as published [20]. Briefly, 1 × 10^4^ cells were cultivated for 10 days in murine embryonic fibroblast conditioned medium supplemented with FGF (25 ng/ml) and Heparin (25 ng/ml) (both from R&D Systems, Wiesbaden, Germany). The medium was exchanged every two days.

### Generation of SOX2-deficient cells by CRISPR/Cas9

To delete the *SOX2* gene in TCam-2 cells, we transfected TCam-2 cells simultaneously with the pX330 vector encoding for three different guide RNAs (gRNA) directed towards the SOX2 coding region using FuGeneHD (Promega, Mannheim, Germany) (transfection ratio 5 : 1; μl FuGeneHD : μg pX330) (Figure S1 A, Table S2) and a GFP-coding plasmid (transfected at a 10× lower concentration compared to the pX330 vector). Two days post transfection, GFP-positive clones, which presumably have taken up the pX330 plasmids were identified, manually picked and clonally expanded.

### DNA, RNA and protein isolation

DNA, RNA and proteins were isolated as described previously [10]. DNA was isolated by phenol/chloroform/isoamylalcohol, RNA by TRIzol and proteins by RIPA buffer.

### Quantitative RT-PCR

Quantitative RT-PCR (qRT-PCR) was performed as described previously [10]. At the end of each PCR run, a melting point analysis was performed. *GAPDH* was used as housekeeping gene and for data normalization. In each qRT-PCR, the TCam-2-ΔSOX2 clones were analyzed in 5 biological replicates and each replicate was analyzed in 3 technical replicates.

### Immunohistochemistry

Immunohistochemistry (IHC) was performed as published previously [10]. Tumor tissues were dissected, fixed in 4 % formalin overnight and processed in paraffin wax. Signal detection was performed semiautomatically in the Autostainer 480 S (Medac, Hamburg, Germany). Nuclei were stained by hematoxylin. See Table S1 for antibody details and dilution ratios. For IHC, 5 tumor tissues from TCam-2-ΔSOX2 clones and 1 tumor tissue from 2102EP cells as control was analyzed.

### Co-immunoprecipitation (Co-IP)

Co-IP was performed using Dynabeads Protein G beads (Life Technologies, Darmstadt, Germany) according to the manual. For each Co-IP reaction, 1.5 mg Dynabeads and 10 μg IP-antibody were used. Antibody binding was performed at room temperature (RT) for 40 minutes (min). Immunoprecipitation of target antigen was performed at RT for 2 hours (h). 200 μg of total protein lysate were used. 10 % of the whole protein lysate (20 μg) were used as input control in western blotting. For elution, the Dynabead-protein-complexes were re-suspended in 20 μl western blot loading buffer (1 × Lämmli buffer in aqua. dest.) and incubated at 95°C for 5 min. Afterwards, Dynabeads were removed by a magnet and eluted samples were analyzed by western blotting. See Table S1 for antibody details.

### Chromatin-immunoprecipitation (ChIP)

For ChIP, the ‘SimpleChIP Enzymatic Chromatin IP Kit (magnetic Beads)’ (Cell Signaling Technology, via NEB, Frankfurt a. M., Germany) was used according to the protocol. Briefly, 1 × 10^7^ cells were cross-linked by formaldehyde and chromatin was sheared enzymatically. For each ChIP, 30 μg Dynabeads and 5 μg of the SOX2 antibody were used. As control, an antibody against rabbit IgG was included. 2% of sheared chromatin was used as input control. Antibody binding to target complexes was performed overnight at 4°C under constant agitation. Isolation of antibody-target-complexes by Dynabeads was performed at 4°C for 2 h under rotation. Afterwards, DNA was reverse-crosslinked, cleaned up by spin columns (included in the ChIP kit) and amplified with the ‘GenomePlex Single Cell Whole Genome Amplification Kit’ (Sigma Aldrich). Amplified DNA was purified by PCI precipitation and analyzed by qPCR. In qPCR, oligonucleotides were used to amplify a PCR-fragment around the SOX2 binding site (on target) and a PCR-fragment within the same gene, but without a SOX2 binding site (off target). Each qPCR analysis was performed in 3 technical replicates. For antibody and primer details see Tables S1 and S2.

### Quantification of DNA methylation levels

5mC levels were quantified as described previously [8]. For quantification, the ‘MethylFlash Methylated DNA Quantification Kit (Colorimetric)’ (Epigentek, via BioCat, Heidelberg, Germany) was used. The experiment was performed according to the manual. For each measurement, 200 ng of genomic DNA were analyzed. Each sample was analyzed in 8 technical replicates. DNA methylation levels were calculated according to the manual using the relative quantification method. For calculation and normalization a positive (methylated polynucleotide containing 50 % 5mC; *n* = 3) and negative (unmethylated polynucleotide containing 50 % cytosine; *n* = 3) control provided in the kit were included.

### Xenotransplantation of GCC cell lines

Xenotransplantation was performed as described previously [10, 30]. 1 × 10^7^ cells in 500 μl of 4°C cold Matrigel (BD, Heidelberg, Germany) were injected into the flank of CD1 nude mice. For 1 week of *in vivo* growth, 1 mouse each was xenografted with TCam-2-ΔSOX2 or parental TCam-2 cells. For 6 weeks of *in vivo* growth, 5 mice were xenografted with TCam-2-ΔSOX2 clones (1–5) and 1 mice each was xenografted with parental TCam-2 or 2102EP cells.

### Illumina HT-12v4 expression microarray

The Illumina expression microarray analysis was performed as published [10]. Samples were processed on Illumina's (San Diego, CA, USA) human‚ HT-12v4′ bead chips. All data were analyzed using‚ Bioconductor R’ (www.bioconductor.org). A subset quantile normalization approach developed by N. Touleimat and J. Tost was applied [32]. Expression values were quantile normalized using the limma’ software package (‘Linear Models for Microarray Data’, www.bioconductor.org). 6 tumor tissues from TCam-2-ΔSOX2 clones (1 after 1 week, 5 after 6 weeks), 2 tumor tissue from parental TCam-2 (1 after 1 week, 1 after 6 weeks), 1 from 2102EP grown for 6 weeks and 1 sample from *in vitro* cultivated TCam-2 was analyzed. Microarray data is publically available via GEO (ncbi.nlm.nih.gov/geo/) (GSE79065).

### Affymetrix expression microarray analysis of GCC tissues

The whole procedure has already been published [21]. The array was reanalyzed in context of this study. In total, 4 seminoma and 7 non-seminoma tissues (teratoma, mixed non-seminoma) were analyzed. To identify genes differentially expressed between seminomas and non-seminomas, normalized, Log_2_-transformed and averaged gene expression intensities of non-seminoma tissues were substracted from averaged seminoma tissues. All genes with a fold change ≥ Log_2_1.5 were considered as significantly deregulated (Data S1 D).

### STRING analysis and Venn diagrams

STRING protein-interaction-prediction were performed online using default settings (string-db.org) [33]. Venn diagrams were generated using ‘Venny’ (bioinfogp.cnb.csic.es/tools/venny).

## SUPPLEMENTARY MATERIALS FIGURES AND TABLES






