# Supplementary material for: SOX2 is essential for in vivo reprogramming of seminoma-like TCam-2 cells to an embryonal carcinoma-like fate
Source: Oncotarget. 2016 Jun 7;7(30):47095–110. doi: 10.18632/oncotarget.9903 (PMC5216926; doi:10.18632/oncotarget.9903)
Supplement: Supplementary file 1 [file oncotarget-07-47095-s001.pdf]

SOX2 is essential for *in vivo* reprogramming of seminoma-like TCam-2 cells to an embryonal carcinoma-like fate

Supplementary Materials

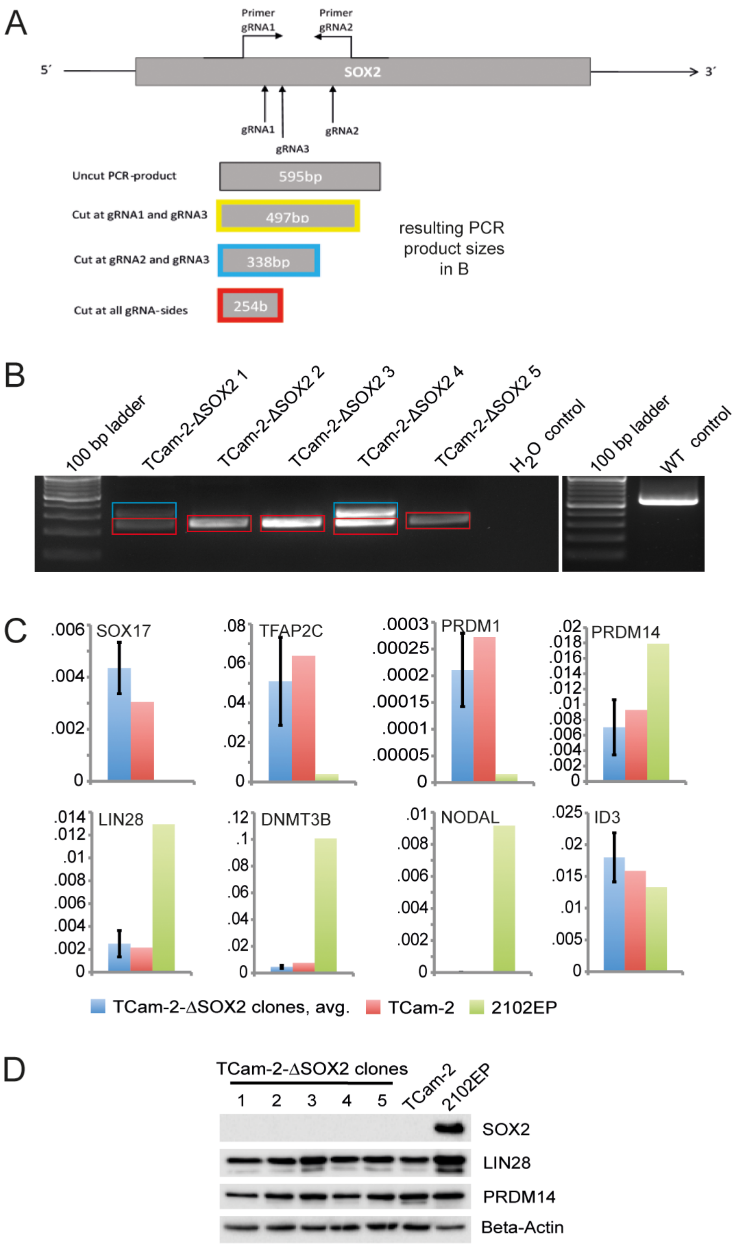

Supplementary Figure S1: (**A**) Illustration of the *SOX2* gRNA target sites within the *SOX2* genomic sequence and different fragment sizes that will be amplified by indicated primers in a PCR reaction depending on the pattern of gRNA targeting. (**B**) PCR analysis of the *SOX2* locus in TCam-2-ΔSOX2 clones. Untranfected TCam-2 served as control (WT control). TCam-2-ΔSOX2 clones 1 and 4 were targeted by all three gRNAs on one allele (red boxes) and by gRNA2 and gRNA3 (blue boxes) on the other allele. TCam-2-ΔSOX2 clones 2, 3 and 5 were targeted by all three gRNAs on both alleles. (**C**) qRT-PCR analysis of indicated pluripotency, seminoma and differentiation markers in parental and SOX2-depleted TCam-2 cells. (**D**) Western blot analysis of indicated proteins in TCam-2-ΔSOX2-clones *in vitro*. Parental TCam-2 and 2102EP cells served as controls.

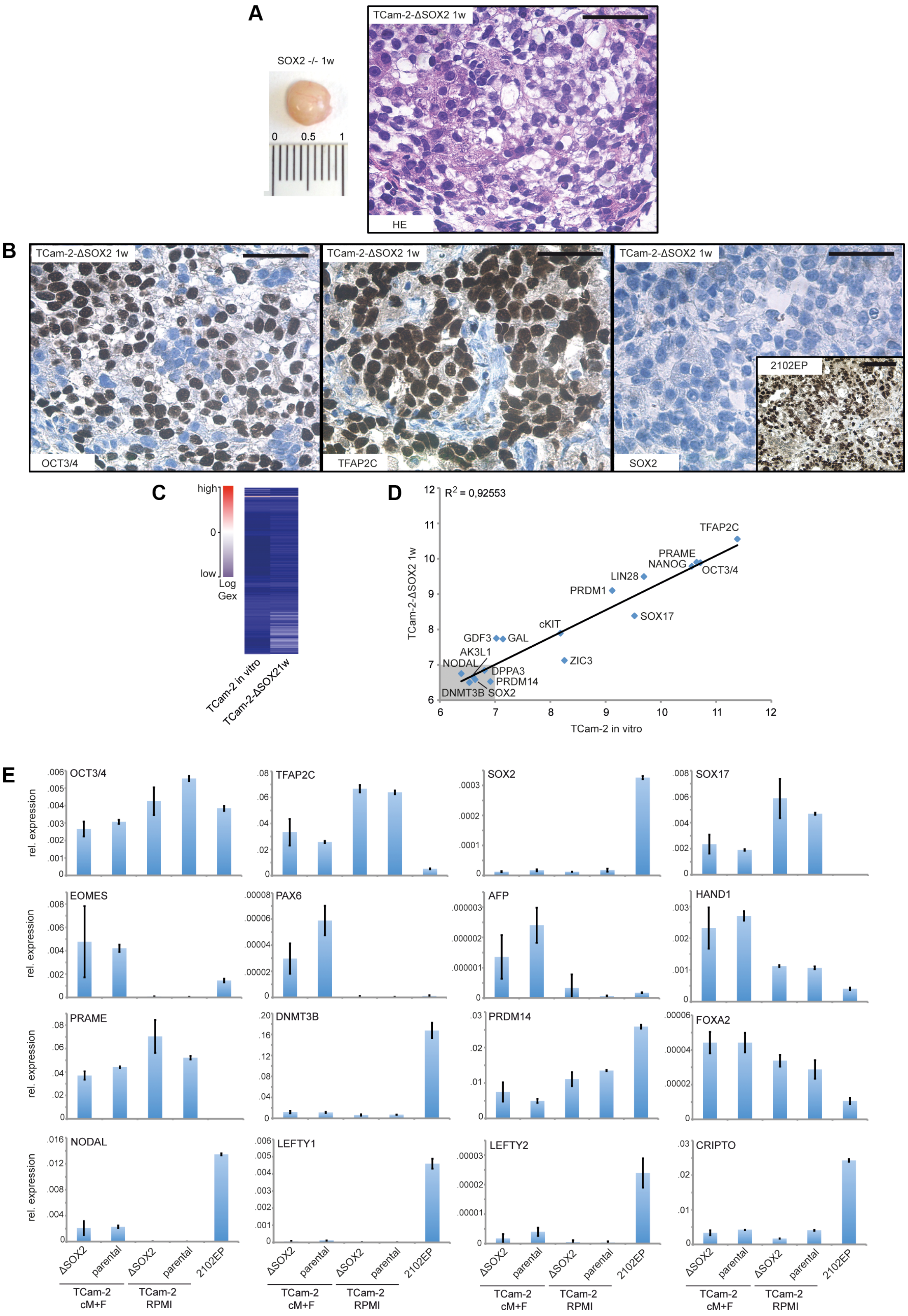

Supplementary Figure S2: (**A**) Macroscopical appearance and HE staining of a tumor from a TCam-2-ΔSOX2 clone grown for 1 week *in vivo*. Scale bar: 50 µm. (**B**) IHC staining of indicated markers in TCam-2-ΔSOX2 tumor tissue 1 week after xenografting. Xenografted 2102EP served as control. Scale bars: 50 µm. (**C**) Heatmap comparing the global expression profiles of *in vitro* cultivated TCam-2 and the TCam-2-ΔSOX2 clone one week after xenografting. (**D**) Comparison of expression intensities of indicated seminoma- and EC-markers in a TCam-2-ΔSOX2 clone 1 week after xenografting and TCam-2 *in vitro*. Genes with expression intensities within the grey area can be considered as not expressed. (**E**) qRT-PCR analysis of indicated genes in TCam-2 and TCam-2-ΔSOX2 cells during cultivation in murine embryonic fibroblast conditioned medium supplemented with FGF4 and heparin (cM+F) or standard culture conditions (RPMI). 2102EP served as EC control.

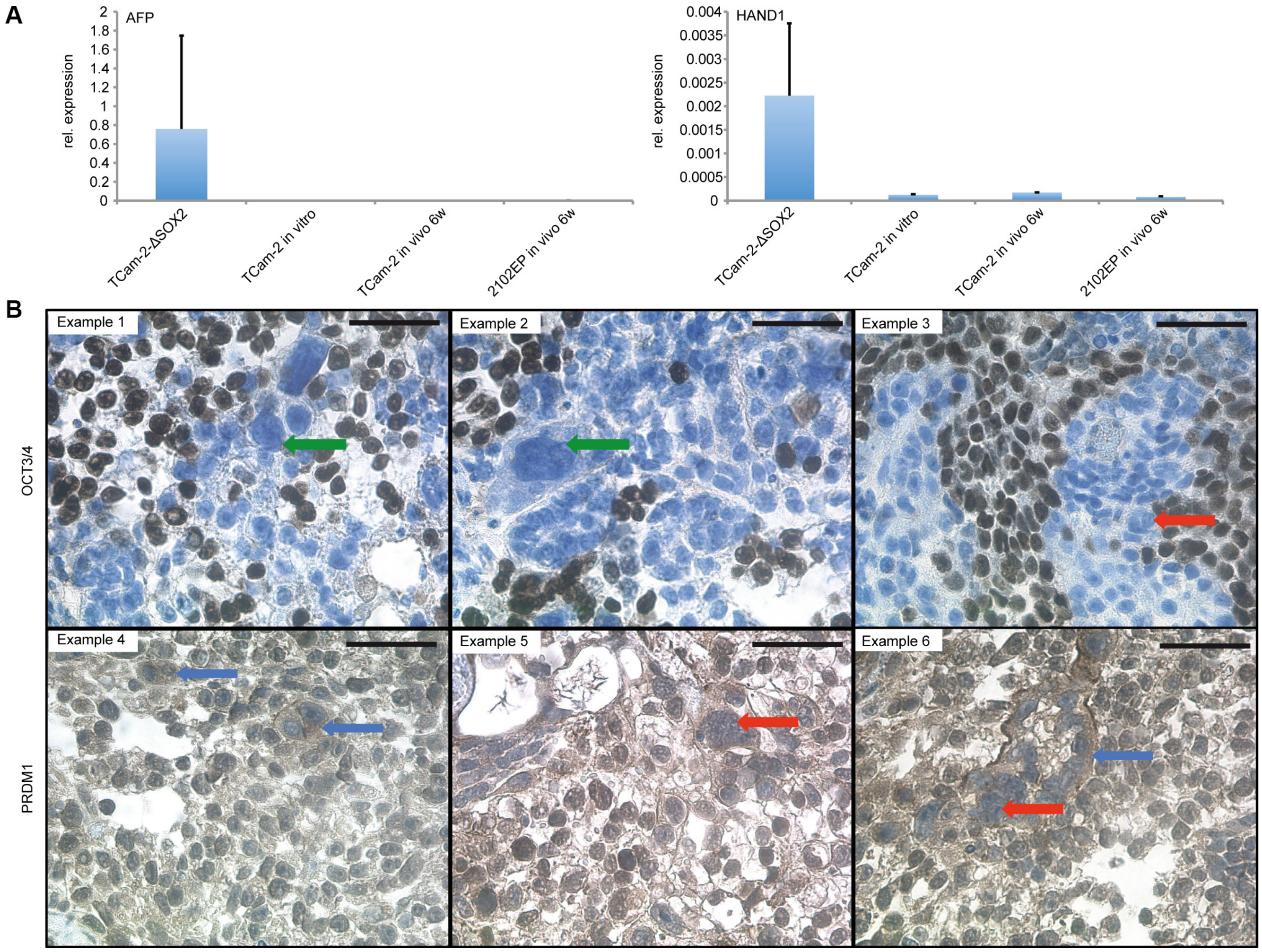

Supplementary Figure S3: (**A**) qRT-PCR analysis of *AFP* and *HAND1* expression in indicated samples. (**B**) IHC staining of inidated markers in TCam-2-ΔSOX2 tumor tissues 6 weeks after xenografting. Green arrows point at cells strongly increased in size, red arrows point at multinucleated cells and blue arrows point at cells showing nuclear exclusion of PRDM1. Scale bars: 50 µm.

Supplementary Data S1: (**A–E**) Expression microarray data and evaluation. See Supplementary_Data_S1

Supplementary Table S1: Antibodies used in this study

Antibody	Company	Clone/Order No.	Immuo	Co-IP	ChIP
AFP	Active motif	39705	1:200	-	-
EOMES	Abcam	ab23345	1:200	-	-
FOXA2	R&D	AF2400	1:200	-	-
H4R3me2	Active Motif	M-20	1:500	-	-
Ki67	Sigma-Aldrich	AB9260	1:200	-	-
OCT3/4	Santa Cruz	C-10	1:200	-	-
PRDM1	H.M. Jäck	-	1:200	-	-
Rabbit IgG	Cell Signaling	2729	-	-	5 microg
SOX17	Abcam	3B10	1:400	10 microg	10 microg
SOX2	R&D systems	AF2018	1:200	-	-
SOX2 (ChIP)	Abcam	ab59776	-	-	5 microg
TFAP2C	Santa Cruz	6E/4	1:200	-	-

Supplementary Table S2: Oligonucleotides used in this study. See Supplementary_Table_S2
